# Designing a provider-led multimorbidity care model for fragmented insurance-based health systems: A mixed-methods study in Colombia

**DOI:** 10.1371/journal.pone.0355752

**Published:** 2026-09-02

**Authors:** Omaira Valencia, Oscar Bernal

**Affiliations:** 1 Faculty of Medicine, Doctoral Program in Public Health, Universidad El Bosque, Bogotá, Colombia; 2 Population Health Division, Fundación Santa Fe de Bogotá, Bogotá, Colombia; 3 School of Government, Universidad de los Andes, Bogotá, Colombia; University of Warwick, UNITED KINGDOM OF GREAT BRITAIN AND NORTHERN IRELAND

## Abstract

Multimorbidity the coexistence of two or more chronic conditions is a growing challenge for health systems in low- and middle-income countries (LMICs) structured around single-disease paradigms. In Colombia, insurer–provider fragmentation within the General System of Social Security in Health (SGSSS) compounds this challenge, generating discontinuities in care that disproportionately affect people living with multiple chronic conditions. Despite a national epidemiological characterisation, a World Bank-supported management proposal, and a formal pilot implementation, limited published evidence exists of a sustained, provider-level, evidence-grounded model for multimorbidity care in fragmented, insurance-based systems. A sequential exploratory mixed-methods design was employed, comprising three phases: (1) a structured evidence synthesis to identify operational domains and implementation gaps in multimorbidity care models; (2) evidence-informed conceptual model construction; and (3) expert feasibility consultation using elements of the Nominal Group Technique with healthcare professionals and system stakeholders (n = 11). Phases were sequentially integrated, with each informing the development of the next. Evidence synthesis identified five recurring structural domains and four cross-cutting implementation gaps, which together informed the construction of the Integrated Provider-level Adaptive Multimorbidity Model (IPAM). The IPAM comprises five interdependent provider-level components designed to function within fragmented, insurance-based systems. Expert consultation confirmed operational feasibility (mean 4.3/5), clinical relevance (4.7/5), and territorial adaptability (4.6/5) of the proposed model. The IPAM offers a structured, adaptable conceptual framework for strengthening multimorbidity care within provider institutions (IPS) operating in fragmented, insurance-based health systems. Its core design principles minimum-viable risk stratification, provider-level governance, proactive follow-up, and graduated technology integration — are transferable to analogous LMIC contexts. Prospective implementation and cost-effectiveness evaluation represent the essential next phase of validation.

## Introduction

Multimorbidity, defined as the coexistence of two or more chronic conditions in the same individual, is a defining challenge for contemporary health systems globally [1,2]. Its prevalence is increasing across LMICs, driven by epidemiological transition and population ageing, and is associated with higher mortality, reduced quality of life, greater treatment burden, and disproportionate growth in healthcare utilisation and costs [3,4]. Health systems remain predominantly organised around single-disease paradigms, creating a structural misalignment with the epidemiological reality faced by a growing proportion of the population.

International frameworks advocate reorientation towards integrated, coordinated models of care [5], and integrated care scholarship identifies alignment across clinical, organisational, and governance levels as a prerequisite for effectiveness [6]. However, evidence on how to operationalise such models in fragmented, insurance-based systems — where financing, risk management, and service provision are institutionally separated — remains limited. A recent synthesis of multimorbidity care models in LMICs confirmed that context-specific, provider-level frameworks are largely absent from the published literature, and that fragmented services and weak governance infrastructure are the dominant implementation barriers [7].

In Colombia, a national assessment documented that approximately 47% of chronic care users within the SGSSS had multimorbidity, with high utilisation, fragmented care pathways, and limited institutional readiness for coordinated management [8]. A subsequent World Bank pilot demonstrated proof of concept for a case manager-based approach but revealed critical sustainability limitations: the model depended on dedicated financing not embedded in standard contracting and did not achieve IPS-level process standardisation [9]. This study addresses that gap by developing and assessing the feasibility of a provider-level, evidence-grounded multimorbidity care model designed for fragmented, insurance-based health systems, using Colombia as the primary development context.

## Methods

### Study design

This study is a sequential exploratory mixed-methods study aimed at developing and assessing the feasibility of a provider-level multimorbidity care model [10,11] comprising three interrelated phases: (1) Evidence synthesis to inform model development; (2) evidence-driven model construction; and (3) expert feasibility consultation. Integration occurred progressively across phases: review-derived domains formed the structural foundation of the model, model construction formalised the component architecture, and expert consultation assessed feasibility and adaptability in the target implementation context. The objective was model development and feasibility assessment, not effectiveness testing.

### Phase 1: Evidence synthesis to inform model development

To inform model development, we conducted a structured evidence-synthesis phase aimed at identifying operational domains, implementation approaches, and recurrent gaps in multimorbidity care models relevant to service delivery redesign in fragmented health systems [12,13]. This phase was designed to support conceptual model construction rather than to produce a standalone scoping or systematic review.

Searches were conducted between May and October 2024 in PubMed/MEDLINE, Scopus, Web of Science, and Google Scholar, covering publications from January 2000 to October 2024 in English and Spanish. An updated search was undertaken in November–December 2025 to identify additional relevant publications through that date. Structured grey-literature searches were also performed in repositories of the WHO, World Bank, PAHO, and the Colombian Ministry of Health. Colombian World Bank institutional reports were included as contextual evidence sources given their direct relevance to the study setting and research objective [8,9,14,15].

Titles, abstracts, and full texts were screened against predefined eligibility criteria (S1 Fig) by two researchers independently; eligibility decisions were documented and discrepancies resolved through discussion. Screening decisions were documented and resolved through iterative review against the study protocol. Data extraction was performed by the primary author using a structured template capturing governance arrangements, stratification mechanisms, care coordination processes, team configuration, information systems, and evaluation focus; extracted data were reviewed in full by a second researcher to verify completeness and consistency. Records were managed in Rayyan.

The synthesis used a framework-mapping approach [12]. Extracted elements were coded deductively against established integrated care frameworks, including the WHO Integrated People-Centred Health Services framework, the JA-CHRODIS Integrated Multimorbidity Care Model, and the Valentijn integrated care typology [5,6,16], while allowing inductive identification of context-specific themes emerging from the included sources. Coding outputs were compared iteratively, and discrepancies were resolved through protocol-guided review. This process generated a final set of structural domains and cross-cutting implementation gaps that directly informed model construction. Domains were further refined through cross-model comparison of European, Latin American, and Colombian sources. Of the 53 sources meeting eligibility criteria, all contributed to domain identification and gap characterisation through framework-mapping. Seventeen evidence entries — drawn from 14 distinct source references with direct, prospectively determined traceability to specific IPAM component design decisions — are presented in the traceability matrix (S1 Table). The criterion for inclusion in S1 Table was whether a source informed a specific component specification, rather than contributing solely to a thematic domain characterisation or a corroborating finding. The remaining sources informed the identification of structural domains and cross-cutting implementation gaps and are cited in the main text and reference list accordingly.

Because the purpose of this evidence-synthesis phase was to identify design-relevant domains and implementation gaps to support model development, rather than to estimate effect sizes or formally assess intervention effectiveness, a formal critical appraisal of individual sources was not undertaken [13].

### Phase 2: Model construction

Model construction translated evidence-derived domains into a conceptual provider-level care framework using abductive logic – iterative movement between evidence, contextual findings, and theory to generate and refine design propositions [10]. The Consolidated Framework for Implementation Research (CFIR) [17] informed identification of modifiable inner-setting constraints relevant to IPS-level implementation. Scoping-derived domains and cross-model gaps were mapped to IPS-modifiable processes and organised into model components based on their functional purpose, responsible actors, and minimum expected process outputs. The resulting component specification informed subsequent expert feasibility consultation.

The Integrated Provider-level Adaptive Multimorbidity Model (IPAM) comprises five interdependent components. The model does not propose reform of financing or insurance architecture; rather, it positions the IPS as an operational first-mover unit by reorganising internal processes, clarifying roles, formalising coordination, and embedding proactive follow-up. Cross-level communication with other providers, insurers, and community networks is defined as a medium-term objective, contingent on the maturation of governance capacity and enabling technology.

### Phase 3: Expert feasibility consultation

The preliminary IPAM underwent structured feasibility consultation through an in-person workshop involving 11 stakeholders: (1) general practitioner and (1) nurse with multimorbidity experience, (2) public health professionals, (3) health administrators, (2) Health services Quality experts and (2) EPS representatives from both contributory and subsidised insurance regimes. The workshop was framed as an expert consultation rather than a formal validation exercise, consistent with the purposive sample size and the developmental stage of the model and was conducted in April 2025.

The workshop incorporated structured elements of the Nominal Group Technique (NGT) [18,19] to support balanced participation and transparent prioritisation. Participants independently reviewed IPAM components and assessed their applicability within the current EPS–IPS context, drawing on evidence-synthesised implementation gaps and their professional experience across clinical, administrative, and insurer roles. A facilitated discussion elicited feasibility considerations, contextual constraints, and suggested refinements for each component. Priorities were agreed through structured group deliberation across two NGT cycles. Proceedings were captured in structured minutes; synthesised feedback informed component refinements without altering the five-component architecture.

Component-level ratings were collected on a five-point Likert scale across three dimensions: operational feasibility, clinical relevance, and territorial adaptability. Mean scores and ranges were calculated per component and overall; interquartile range was reported selectively where score dispersion exceeded the modal response, providing additional distributional information without assuming normality. Modifications requested during deliberation were documented and mapped to the corresponding component.

### Ethical considerations

This study did not involve primary data collection from human participants beyond expert stakeholders who participated as professional informants in a service design consultation. This study was approved by the Ethics Committee of Fundación Santa Fe de Bogotá (Communication 16596, June 2024). The expert consultation was conducted with the informed agreement of all participants and informed consent was obtained through signed attendance records, which documented their voluntary agreement to participate and to allow the use of anonymised information generated during the consultation. No identifiable personal data were collected or retained. The consultation did not involve patients. All sources used in the evidence-synthesis phase were publicly available.

### Reporting

The overall study is reported as a sequential exploratory mixed-methods study. The evidence-synthesis component is described in sufficient detail to ensure transparency and reproducibility, the expert consultation is reported following NGT reporting conventions [18,19].

## Results

### Findings from the evidence-synthesis phase

The final sample comprised 53 sources: 29 peer-reviewed publications (including three identified through the updated search), 16 grey literature documents, and eight institutional reports (S1 Fig). The evidence synthesis yielded 53 sources meeting eligibility criteria across five structural domains: (1) population-based risk stratification; (2) care delivery redesign with multidimensional assessment and medication reconciliation; (3) governance and communication mechanisms; (4) proactive follow-up and self-management support; and (5) enabling technology with graduated implementation. Four cross-cutting implementation gaps were consistently identified: absence of multimorbidity-specific stratification tools adapted for IPS-level use; lack of standardised individualised care plan (ICP) documentation; weak provider-level governance infrastructure; and technology heterogeneity incompatible with high-entry-point requirements. Table 1 presents the key models identified and their principal characteristics. Additional evidence on care model elements and implementation gaps in LMIC settings was provided by two targeted systematic reviews included in the updated search [7,33,34].

**Table 1:** Key multimorbidity care models identified in the integrated scoping review.

Model/ Source	System context	Risk stratification	Core components	Governance arrangements	Key limitations/ gaps
**JA-CHRODIS IMCM** [6,20–22]	Europe (11 countries); universal/mixed systems	Risk-based validated tools (interRAI, CIRS); case-mix classification	MDT, individualised care plans, care coordination, self-management, transition care	Institutional protocols; cross-sector integration; shared governance framework	Sustainability limited after project funding; not designed for insurer–provider fragmented systems
**Catalan PPAC/MACA** [23]	Catalonia, Spain; universal single-payer	Complexity and frailty stratification; population registers	Person-centred advanced care planning; shared documentation; MDT	Formal governance; integrated regional IT system	Highly dependent on robust IT and single-payer context; not transferable to fragmented settings
**SELFIE European mapping** [24]	9 European countries; predominantly universal	Variable; risk scoring + clinical criteria	Case management, self-management, coordination mechanisms	Limited governance formalisation; highly variable across sites	No LMIC application; limited rigorous outcome evaluation; variable governance
**WB Colombia proposal** [8,14]	Colombia; SGSSS (EPS + IPS)	Risk assessment within EPS; variable tools	Case management; caregiver support; coordination protocols; psychosocial component	EPS-driven; limited IPS-level governance formalisation	Designed at EPS level, not IPS; not sustained beyond project period
**Chilean MACEP** [25–29]	Chile; universal Fonasa public system	Population-level ACG tool; centralised administrative databases	High-risk registry, personalised care plan, interdisciplinary team, proactive contact, transitional care	Embedded in national primary care reform; EHR-integrated at system level	ACG requires centralised databases unavailable in most LMICs; results may not transfer to fragmented systems
**WB Colombia pilot** [9,15]	Colombia; 4 EPS; multiple IPS settings	IPS-level identification using clinical criteria	Case manager; home visits; patient education; caregiver integration; MDT coordination	Institutional coordination protocol; monthly case review; no formal indicator framework	Case manager dependency; absent ICP/reconciliation standards; technology barriers across IPS; no cost-effectiveness data
**Mesa-Melgarejo et al.** [30]	Colombia; case management; mixed methods	Clinical identification by nurses	Nurse case management; patient education; medication review; caregiver support	Nurse-led within IPS	Limited to nurse-led model; no broader IPS redesign; short follow-up
**South Africa ICDM** [31,32]	South Africa; mixed public primary care; district health authority	Chronic disease registers; condition-based targeting	Integrated chronic disease management; community health workers; standardised protocols; clinical mentoring	Facility-level committees; national DoH guidelines; district support teams	Limited to public facilities; implementation fidelity variable; no MM-specific stratification; sustainability challenges beyond project periods

MDT = multidisciplinary team; ICP = individualised care plan; ACG = Adjusted Clinical Groups; LMIC = low- and middle-income country; EPS = health insurance entity; IPS = institution providing health services; MM = multimorbidity; WB = World Bank; DoH = Department of Health; ICDM = Integrated Chronic Disease Management; IPAM = Integrated Provider-level Adaptive Multimorbidity Model.

### IPAM framework

Evidence synthesis informed construction of the IPAM. The model is organised around five interdependent components within three domains: (1) population-based management and risk stratification; (2) clinical coordination and continuity; and (3) governance and system integration at provider level. Territorial adaptability is a cross-cutting design criterion. The IPAM is designed as a sequential first step: IPS-level process standardisation (C1–C3) creates the organisational foundation upon which proactive follow-up (C4) and cross-level communication enabled by technology (C5) become operationally achievable. This sequencing responds directly to the World Bank pilot’s finding that coordination mechanisms not rooted in institutional process redesign cannot be sustained [9,15]. Table 2 maps each component to its evidence base and the specific gap addressed.

**Table 2:** IPAM components: evidence base, gaps addressed, and World Bank Colombia pilot findings.

IPAM component	International and Colombian evidence base	Gap addressed	WB pilot specific finding
**C1: Population-based health management and risk stratification**	JA-CHRODIS IMCM [6,20–22]; SELFIE [24]; MACEP [25–27]; Varela et al. [28]; Sapag et al. [29], WHO IPCHS [5]; WB national assessment [8]; WB proposal [14]	No MM-specific stratification within Colombian IPS [7,33,34]; EPS-level identification not accessible to IPS; IT heterogeneity requires adaptable minimum-viable criteria	Pilot relied on EPS-level identification with limited IPS tools; technology barriers in multiple sites [9]
**C2: Care delivery redesign — multidimensional assessment, medication reconciliation, ICP co-designed with patients**	Cochrane review [35]; JA-CHRODIS [6,20–22]; Italian MM guidelines [22]; polypharmacy management [36]; WB pilot [9]	Conceptual ambiguity in reconciliation; absent ICP documentation; single-disease encounter structure [7, 33]	Pilot explicitly identified absent ICP and variable reconciliation as care quality barriers [9]
**C3: Governance and communication — structured case conferences, referral pathways, patient–institution channels, MM committee**	JA-CHRODIS governance [6,20–22]; SELFIE [24]; Valentijn typology [16]; WB successful experiences [15]; **OECD/ Yordanov [**37,38**]**	EPS–IPS separation limits system coordination; informal interprofessional communication; absent shared documentation [7,33]	Case manager model unsustainable without IPS-level process design; high-performing IPS shared 3 governance features [15]
**C4: Proactive follow-up and self-management — scheduled outreach, adherence monitoring, deprescribing, caregiver support**	Cochrane [35]; MACEP transitional care [27]; Varela et al. [28]; Sapag et al. [29]; WB successful experiences [15]	Absent standardised follow-up; loss-to-follow-up in dispersed populations; caregiver support absent from IPS protocols [7,33]	Caregiver integration and proactive contact most valued but required unsustained dedicated staffing [15]
**C5: Enabling technology — graduated: registries → interoperability → analytics → telehealth (transversal C1–C4)**	SELFIE [24]; WB pilot technology documentation [9]	Heterogeneous IT capacity; digital literacy barriers; technology cannot be an entry prerequisite in fragmented LMIC settings	Significant technology barriers — including paper-based records — excluded lower-capacity IPS from effective participation [9]

*ICP = individualised care plan; MM = multimorbidity; WB = World Bank; IPS = institution providing health services; EPS = health insurance entity, IT = information technology; LMIC = low- and middle-income country.*

### Component 1: Population-based health management (C1)

C1 establishes systematic identification and risk stratification of multimorbidity patients within IPS. Unlike MACEP’s centralised ACG-dependent analytics [25–27] or the World Bank pilot’s EPS-level identification [9], the IPAM defines minimum stratification criteria adaptable to local data capacity: multimorbidity burden, functional status, and frailty assessment for adults ≥65.

The selection of these three criteria reflects a convergence of epidemiological evidence, evaluated model design, and contextual feasibility constraints [39]. Multimorbidity burden — operationalised as the number of concurrent chronic conditions — is the most consistently documented predictor of high healthcare utilisation, care complexity, and adverse outcomes across LMIC settings [1,3,7]; Oliveira et al. confirm that condition count remains the most operationalisable stratification criterion in primary care settings with limited data infrastructure [34]. Functional status is included because disability and functional decline are the primary mediators between multimorbidity burden and high-cost care trajectories [3], and because functional assessment does not require centralised databases or validated population-level tools — both of which are unavailable across a substantial proportion of Colombian IPS [9,34]; evaluated models including JA-CHRODIS and the Cochrane synthesis identify functional assessment as a key stratification variable and moderator of intervention effectiveness [6,20,35]. Frailty assessment for adults ≥65 is included as a third criterion because the co-occurrence of frailty and multimorbidity amplifies risk of functional dependence, unplanned hospitalisation, and care fragmentation disproportionately in this age group [3]; its inclusion as a mandatory stratification component is explicitly recommended in the Italian multimorbidity guidelines [22] and operationalised in the JA-CHRODIS implementation framework [20]. Taken together, these three criteria identify patients at highest risk of high-need, high-cost trajectories while remaining implementable with paper-based registries at IPS entry level — a design constraint directly informed by the technology barriers documented in the World Bank pilot [9] and the centralised-database dependency that limits the transferability of ACG-based tools to fragmented LMIC contexts [25,26,34].

Geolocation and territorial mapping enable prioritisation of outreach for dispersed or socially vulnerable populations, responding to the geographic disparities documented in the national assessment [8]. This component does not require IT infrastructure beyond a structured patient registry, which may be paper-based at entry level.

### Component 2: Care delivery redesign (C2)

C2 formalises clinical process redesign within IPS. It mandates structured multidimensional assessment, systematic medication reconciliation with defined operational steps, and documented individualised care plans (ICP) co-designed with patients using shared decision-making. Evidence synthesis and gap analysis identified conceptual ambiguity in reconciliation and absent ICP documentation as consistent cross-model gaps [7,33]; the World Bank pilot identified the same as direct barriers to care quality [9]. C2 specifies reconciliation as a distinct clinical procedure — documented in the health record — and the ICP as a mandatory output of initial multimorbidity assessment. Interdisciplinary roles are clarified, consultation workflows reorganised for comprehensive assessment, and structured case conferences institutionalised for complex cases.

### Component 3: Governance and communication (C3)

C3 addresses the governance gap exposed by the World Bank pilot: when coordination depends on a single case manager role rather than institutional process design, sustainability collapses when dedicated staffing is withdrawn [9,15]. C3 formalises provider-level governance through: interprofessional case conferences (minimum monthly), shared care documentation with all specialist referrals, direct patient–institution communication channels, an institutional multimorbidity committee with assigned resources, and performance monitoring using the Table 3 indicator matrix. Communication pathways with other levels of care are defined as structured objectives, building progressively as institutional capacity matures. The evidence from high-performing IPS in the pilot [15] — where protected MDT time, systematic registries, and direct patient communication were the distinguishing features — is directly operationalised here as minimum standards.

**Table 3:** IPAM performance indicator framework (structure–process–outcome logic; Donabedian [40]).

Component	Indicator	Type	Frequency	Data source/ method
**C1: Population-based health management**				
C1	% patients with MM risk stratification completed	Process	Quarterly	EHR/ institutional registry
C1	% adults ≥65 with frailty screening completed	Process	Quarterly	EHR/ geriatric records
C1	Community resources mapped per territory (n)	Structure	Annual	Territorial mapping database
C1	Intersectoral referral rate per 1,000 MM patients	Process	Semi-annual	Referral management system
**C2: Care delivery redesign**				
C2	% MM patients with documented ICP	Process	Quarterly	EHR audit
C2	% staff trained in MM protocols and shared decision-making	Structure	Annual	Training records
C2	% consultations with documented medication reconciliation	Process	Quarterly	EHR completeness rate
C2	Treatment burden — patient-reported (CollaboRATE/ SDM-Q-9)	Outcome	Semi-annual	Patient survey
**C3: Governance and communication**				
C3	Interprofessional case conferences held per month	Process	Monthly	Meeting records
C3	% specialist referrals with shared care plan attached	Process	Quarterly	Referral system audit
C3	Median waiting time for specialist appointment (MM patients), days	Process	Monthly	Appointment management system
C3	% patients reporting accessible direct communication channels	Outcome	Semi-annual	Patient experience survey
**C4: Proactive follow-up and self-management support**				
C4	% MM patients with ≥1 proactive contact in preceding 3 months	Process	Quarterly	Follow-up registry
C4	Concordance between declared and patient-perceived follow-up	Outcome	Semi-annual	Dual audit
C4	Medication adherence rate (self-report + dispensing data)	Outcome	Quarterly	Self-report + pharmacy data
C4	% eligible patients with deprescribing review completed	Process	Semi-annual	EHR/ prescription audit
C4	Caregiver burden index (Zarit modified)	Outcome	Annual	Caregiver survey
**C5: Enabling technology**				
C5	% follow-up contacts conducted via telehealth	Process	Quarterly	Telehealth system data
C5	Digital tool usability satisfaction (SUS scale)	Outcome	Annual	Patient survey
**Global/ system-level outcome indicators**				
Global	Avoidable emergency visits/ 1,000 MM patients/ year	Outcome	Annual	RIPS administrative data
Global	Unplanned hospitalisations/ 1,000 MM patients/ year	Outcome	Annual	RIPS administrative data
Global	Health-related quality of life (EQ-5D-5L)	Outcome	Annual	PROM — patient survey
Global	Chronic illness care experience (PACIC)	Outcome	Annual	Patient survey
Global	Cost per MM patient per year (direct healthcare costs)	Outcome	Annual	Cost data + RIPS

*MM = multimorbidity; ICP = individualised care plan; EHR = electronic health record; SUS = System Usability Scale; PACIC = Patient Assessment of Chronic Illness Care; RIPS = Registros Individuales de Prestación de Servicios de Salud.*

### Component 4: Proactive follow-up and self-management support (C4)

C4 reconceptualises follow-up as proactive, continuous, and bidirectional. Scheduled outreach, adherence monitoring, deprescribing review, symptom reporting channels, and structured psychosocial and caregiver support are institutionalised within IPS workflows rather than delegated to a dedicated external role. The World Bank pilot demonstrated that caregiver integration and proactive contact were the most valued elements by participants [15]; the MACEP evaluation confirmed proactive contact rate as a predictor of effectiveness [29]. Operational safeguards include verified contact systems and patient registries to prevent loss to follow-up.

### Component 5: Enabling technology (C5)

C5 is explicitly transversal — a cross-cutting enabler that progressively unlocks the coordination potential of C1–C4 rather than a prerequisite that must be satisfied before implementation begins. This component responds directly to the World Bank pilot finding that technology heterogeneity limited participation of lower-capacity IPS [9]. Implementation is graduated across three phases: Phase 1 (minimum viable) accepts paper-based systems; Phase 2 adds EHR templates, telehealth capacity, and drug interaction alerts; Phase 3 includes analytics dashboards and interoperable cross-level records.

### Expert feasibility consultation

Score distributions indicated high consensus across most components and dimensions. C1 and C2 showed the narrowest score ranges (4–5 across all dimensions), reflecting near-unanimous agreement on the operational feasibility and clinical relevance of population-based stratification and care delivery redesign within the IPS context. C3 and C4 operational feasibility scores showed slightly greater dispersion (range 3–5; IQR 4–5 and 4–4, respectively), indicating that while most participants rated these components favourably, a minority expressed moderate reservations regarding governance formalisation and the operational demands of institutionalised proactive follow-up. C5 showed the greatest score dispersion on operational feasibility (range 2–5; IQR 3–4; mean 3.8/5), consistent with participants’ recognition that technology capacity varies substantially across IPS settings; this pattern directly reinforces the rationale for a graduated rather than prerequisite technology implementation approach. Table 4 presents component-level results and score distributions.

**Table 4:** Expert feasibility consultation results by IPAM component (n = 11 stakeholders; Nominal Group Technique).

IPAM component	Operational feasibility mean (range) [IQR**]	Clinical relevance mean (range) [IQR**]	Territorial adaptability mean (range) [IQR**]	Implementation priority	Key modifications requested
**C1: Population-based health management**	**4.7 (4–5)**	**4.6 (4–5)**	**4.8 (4–5)**	**1st**	**Incorporate social determinants; frailty screening ≥65; paper-based registry accepted**
**C2: Care delivery redesign**	**4.5 (4–5)**	**4.9 (4–5)**	**4.6 (4–5)**	**2nd**	**Protected consultation time ≥45 min; structured reconciliation form; clear reconciliation definitions**
**C3: Governance and communication**	**4.3 (3–5)** **[IQR 4–5]**	**4.7 (4–5)**	**4.4 (3–5)** **[IQR 4–5]**	**3rd**	**EPS contracting incentive alignment; monthly case conferences; institutional MM committee**
**C4: Proactive follow-up and self-management**	**4.2 (3–5)** **[IQR 4–4]**	**4.8 (4–5)**	**4.5 (4–5)**	**2nd (tied)**	**Caregiver inclusion; verified contact; community health worker integration**
**C5: Enabling technology**	**3.8 (2–5)** **[IQR 3–4]**	**4.5 (4–5)**	**4.9 (4–5)**	**4th**	**Paper-based fallback; basic registry Phase 1; advanced analytics Phase 3 only**
**Overall IPAM**	**4.3**	**4.7**	**4.6**	**—**	**Unanimous consensus on IPS-led feasibility**

**IQR = interquartile range. The IQR is reported selectively for components and dimensions where score dispersion exceeded the modal response, providing additional distributional information beyond the range. Where the IQR was identical to the observed range or collapsed to a single value (IQR = 5–5), it was omitted as uninformative

*Ratings on a 5-point Likert scale (1 = strongly disagree; 5 = strongly agree). NGT = Nominal Group Technique. Framed as feasibility assessment, not confirmatory validation.*

Three cross-component implementation priorities emerged from NGT deliberation. First, EPS contracting incentive alignment was identified by all participants as the single most critical sustainability enabler, directly consistent with the World Bank pilot’s finding that dedicated case manager financing was unsustainable without explicit EPS commitment [9,15] and with international evidence that value-based payment models require structured provider accountability mechanisms to sustain coordination [37]. Second, protected interdisciplinary team time, currently absent from standard IPS scheduling was unanimously identified as a structural prerequisite for operationalising case conferences and multidimensional assessment workflows. Third, incorporation of social determinants — particularly housing and food insecurity — into C1 stratification criteria was prioritised in response to disparities documented in the national assessment [8].

No component was rated below 3.8/5 on any dimension, and no participant recommended removing any component or substantially altering the five-component architecture.

The traceability matrix mapping each evidence source and implementation gap to the corresponding IPAM design response is provided in Supplementary Table 1 (S1 Table).

## Discussion

The IPAM offers a provider-level conceptual pathway to strengthen multimorbidity care in fragmented, insurance-based systems where payer–provider separation constrains system-wide integration. The model was developed through three sequentially integrated phases evidence synthesis, abductive model construction, and structured feasibility consultation and positions health service providers (IPS) as a pragmatic entry point for improving coordination within their operational remit, while acknowledging that sustainable scale-up will require complementary reforms in contracting, data-sharing, and governance. This framing aligns with international calls to reorient services toward integrated models of care [5], while recognising that effectiveness remains context-dependent and shaped by governance and financing realities [35].

A central contribution of the IPAM is its explicit sequencing logic: strengthening internal process reliability and governance arrangements within IPS creates the conditions under which proactive follow-up and technology-enabled coordination become operationally feasible. This is consistent with Donabedian’s proposition that improvements in structure and process precede measurable outcome change [40], and with the Valentijn integrated care typology, which identifies clinical and professional integration as foundational to broader system integration [16]. In practice, this sequencing addresses a recurrent challenge in fragmented systems: coordination mechanisms introduced as add-ons such as isolated case manager roles without institutional process redesign have limited sustainability when dedicated project resources are withdrawn [9,15]. By embedding coordination expectations into provider governance and routine clinical workflows, the IPAM aims to reduce reliance on short-term project staffing and to support continuity as part of standard care delivery [41].

The IPAM also advances the Colombian evidence base by responding directly to prior institutional experience [42,43]. The World Bank pilot demonstrated proof of concept but identified structural constraints related to sustainability, process standardisation, and heterogeneous information capacity [9,15]. The IPAM addresses these by specifying minimum-viable requirements that permit entry even in low-digitisation environments, while outlining a graduated pathway toward more advanced functionality as governance capacity and enabling technology mature. This design is particularly relevant in settings where infrastructure varies sharply across territories and provider organisations, as documented in the national assessment [8].

Comparison with evaluated models helps clarify both the plausibility and the boundaries of the IPAM. MACEP provides the closest Latin American benchmark, with demonstrated reductions in avoidable hospitalisation [25], favourable cost-effectiveness [26], and implementation evidence highlighting the importance of proactive contact and care plan completion [29]. These findings support prioritising proactive follow-up and documented individual care plans as core functions within C4 and C2 respectively. However, direct transferability is constrained by structural differences: MACEP operates within a more integrated public architecture and relies on centralised risk stratification capacity not uniformly available across Colombian territories [25–27]. The IPAM therefore diverges by design through tool-agnostic stratification, provider-level governance that does not assume payer integration, and a scalable technology approach specifying minimum-viable registries as the entry point. Similarly, JA-CHRODIS demonstrated the value of institutional multimorbidity committees and formalised interprofessional governance, but sustainability was limited in lower-resource governance contexts after project funding ended [6,20–22] a limitation directly addressed by the IPAM’s institutional committee design with assigned resources.

Beyond Colombia and Chile, related integrated chronic disease initiatives in LMIC contexts including South Africa’s Integrated Chronic Disease Management model and evidence on chronic care integration in sub-Saharan Africa, as well as primary care strengthening reforms in Brazil and India highlight similar implementation constraints around workforce capacity, continuity mechanisms, and heterogeneous information infrastructure [31,32,44,45]. A recent synthesis of care models for multimorbidity in LMICs confirmed that context-specific, provider-level frameworks are largely absent from the published evidence base, and that fragmented services and weak governance infrastructure are the dominant barriers to implementation [7]. A scoping review of systematic reviews on integrated care models for multimorbidity further confirmed that intervention components vary widely and their effectiveness remains uncertain across settings, reinforcing the need for context-specific, adaptable frameworks [46]. A complementary review identified lack of multimorbidity-specific guidelines and absent care coordination protocols as recurring platform-level barriers in LMIC primary health systems [33]. These findings reinforce the rationale for the IPAM’s design and the transferability of its principles to analogous fragmented, insurance-based contexts in Latin America, Asia, and sub-Saharan Africa, consistent with international evidence that implementing integrated care at scale requires strategies addressing governance, co-design, and financing alignment simultaneously [47].

The IPAM has implications for policy and contracting in fragmented insurance-based systems. Stakeholder input during the expert consultation and prior Colombian experience consistently identified EPS contracting incentive alignment as the single most critical sustainability enabler consistent with the World Bank pilot’s finding that dedicated case manager financing was unsustainable without explicit payer commitment [9,15]. International evidence indicates that bundled payment or capitation arrangements for defined multimorbidity cohorts can encourage investment in coordination and proactive follow-up, provided that data-sharing and accountability mechanisms are in place [37,38]; however, payment reforms targeting multimorbidity specifically remain scarce and their effects inconsistent, particularly when not combined with multifaceted care delivery redesign [48]. Colombia’s policy frameworks provide a supportive direction, Resolución 229 de 2020 [49] and the Plan Decenal de Salud Pública 2022–2031 [50] but operational translation into routine contracting and accountability remains uneven. In this context, the IPAM’s Table 3 monitoring framework serves a dual purpose: in the short term, it functions as an implementation tool for generating credible process evidence and supporting iterative improvement within IPS, in the medium term, it is designed to provide the pre-specified indicator sets and quality benchmarks that performance-based contracting between IPS and EPS requires consistent with OECD evidence that value-based payment models require structured provider accountability mechanisms, including pre-specified outcome indicators, to hold providers responsible for coordination and care quality [37].

Economic considerations reinforce the priority of prospective evaluation. Cost-effectiveness claims cannot be made on the basis of conceptual design alone, particularly given that the World Bank pilot did not generate comprehensive cost data [9,15]. MACEP provides a methodological precedent for economic evaluation in Latin America using pre-specified outcome endpoints [26], but Colombian unit costs, baseline utilisation patterns, and contractual incentive structures differ meaningfully. Future studies should incorporate prospective economic evaluation with pre-specified endpoints and reporting aligned with CHEERS 2022, alongside sensitivity analyses reflecting ongoing debate about willingness-to-pay thresholds in middle-income contexts [51]. Such evaluation is essential not only for policy uptake but also for clarifying the resource implications of protected interdisciplinary team time, follow-up intensity, and minimum data infrastructure each identified as priority implementation requirements by expert stakeholders.

The expert feasibility consultation, while structured using the Nominal Group Technique, involved a purposive sample of 11 stakeholders and was framed as a developmental assessment rather than confirmatory validation. Patients and caregivers were not represented in the consultation, which is an acknowledged limitation and a priority for subsequent co-design and pilot implementation work. Prospective implementation should incorporate patient and caregiver participation through structured co-design approaches such as experience-based co-design or participatory action research applied specifically to the adaptation of IPAM components to each IPS context. Evidence suggests that co-design improves the relevance, usability, and acceptability of multimorbidity care interventions, and is essential for positioning the IPAM not solely as an organisational model but as a genuinely person-centred one [52]. Future implementation phases should prioritise the prospective operationalisation of the patient-reported outcome measures already defined in the Table 3 indicator framework, consistent with the international consensus that a deeper understanding of care quality requires measuring what matters to people [53]. The evidence-synthesis was limited to English and Spanish sources and may have missed relevant evidence in other languages. Reliance on programmatic institutional reports as primary evidence sources for the Colombian context [9,15] introduces the possibility of reporting bias. Finally, although the IPAM draws on a robust international evidence base, its design principles have been developed for the Colombian SGSSS architecture; adaptation will be required for contexts with substantially different governance, financing, or workforce configurations.

## Conclusion

The IPAM translates international evidence and documented Colombian implementation experience into a structured, provider-level conceptual framework for multimorbidity care in fragmented, insurance-based health systems. The model’s foundational logic is sequential: IPS-level process standardisation (C1–C3) establishes the organisational foundation; proactive follow-up (C4) institutionalises continuity of care; and enabling technology (C5), graduated from minimum-viable registries to advanced interoperable systems, progressively unlocks cross-level coordination with other care providers, insurers, and community networks as institutional capacity develops. This sequencing responds to the structural reality of fragmented systems and to the central lesson of the World Bank pilot: coordination mechanisms not rooted in institutional process redesign cannot be sustained [9,15].

The IPAM’s core design principles tool-agnostic risk stratification, provider-level governance, institutionalised proactive follow-up, and graduated technology are transferable to other LMICs and middle-income contexts sharing fragmented, insurance-based architectures. The next essential steps are prospective pilot implementation using hybrid effectiveness–implementation designs, formal cost-effectiveness evaluation with pre-specified endpoints, and iterative refinement informed by patient and caregiver participation. Until such evaluation is available, the IPAM should be used to guide implementation planning and process monitoring, not to assert proven effectiveness.

## Supporting information

S1 Fig PRISMA-ScR flow diagram. Sources of evidence.
(SVG)

S1 Table Traceability matrix: evidence sources and implementation gaps mapped to IPAM design responses. (DOCX)
