# Supplementary material for: Designing a provider-led multimorbidity care model for fragmented insurance-based health systems: A mixed-methods study in Colombia
Source: PLoS One. 2026 Sep 2;21(9):e0355752. doi: 10.1371/journal.pone.0355752 (PMC13537686; doi:10.1371/journal.pone.0355752)
Supplement: Supplementary file 1 [file pone.0355752.s002.docx]

**SUPPLEMENTARY MATERIAL**

**S1 Table.** Traceability matrix: evidence sources and implementation gaps mapped to IPAM design responses.

| **Evidence source** | **Evidence type** | **Gap identified** | **IPAM component(s)** | **IPAM design response** |
| --- | --- | --- | --- | --- |
| **Cochrane systematic review [34]** | *Systematic review* | All multimorbidity RCTs conducted in high-income settings; no evaluated model for fragmented LMIC systems | **C2, C4** | C2 and C4 design draws on RCT-supported interventions (structured care planning, proactive follow-up) adapted to fragmented LMIC context |
| **WB national assessment [6]** | *Programmatic / institutional report* | ~47% SGSSS chronic care users had MM 2012–2016; high utilisation + fragmented care; geographic and socioeconomic disparities | **C1, C3** | Population-based C1 stratification; C3 governance addresses cross-level fragmentation; social determinants incorporated into stratification criteria |
| **WB Colombia proposal [12]** | *Programmatic / institutional report* | EPS-driven model: EPS–IPS fragmentation identified as central barrier; no MM-specific stratification tools at IPS level; caregiver support underrepresented; IPS-level governance absent | **C1, C3, C4** | C1 stratification repositioned at IPS level with minimum-viable criteria independent of EPS tools; IPAM situates governance at IPS level (not EPS); C4 formally integrates caregiver in follow-up and support protocols |
| **WB pilot [7]** | *Programmatic / institutional report* | Case manager model modest and non-sustained; absent ICP/reconciliation standards; technology barriers across IPS; EPS-level patient identification with limited IPS-level tools | **C1, C2, C5** | C1 defines IPS-level stratification criteria independent of EPS tools; C2 mandates standardised reconciliation and ICP documentation; C5 graduated model with paper-based entry point |
| **WB pilot [7]** | *Programmatic / institutional report* | Fragmented clinical records; no unified health information system across EPS–IPS; paper-based records in several IPS | **C3, C5** | C3 specifies shared care documentation with all specialist referrals; C5 paper-based fallback as Phase 1 entry point |
| **WB successful experiences [13]** | *Programmatic / institutional report* | IPS with strongest outcomes shared three features: protected MDT time, systematic follow-up registry, direct patient–institution communication channels | **C3, C4** | All three features formalised as minimum operational standards in C3 and C4 |
| **WB successful experiences [13]** | *Programmatic / institutional report* | Case manager dependency unsustainable; absent indicator framework for EPS contracting discussions | **C3, Table 4** | Institutional MM committee with assigned resources; Table 4 indicator matrix designed to support EPS contracting evidence |
| **Varela et al. [30]; Sapag et al. [31]** | *Implementation evaluation* | Proactive contact rate and care plan completion identified as key performance predictors in MACEP; caregiver integration most valued by participants | **C1, C4** | C1 registry designed to enable proactive contact tracking; C4 institutionalises proactive outreach and caregiver integration as core workflow elements |
| **Mesa-Melgarejo et al. [27]** | *Observational study* | Nurse case management improved outcomes but limited to single professional role; no broader IPS process redesign | **C2, C3** | C2 clarifies full interdisciplinary role configuration; C3 formalises interprofessional governance beyond single-role model |
| **MACEP [24,25,26]** | *Population study; economic evaluation; implementation evaluation* | ACG stratification tool requires centralised administrative databases unavailable in most Colombian and LMIC territories | **C1** | Minimum-viable stratification criteria adopted (MM burden + functional status + frailty assessment ≥65); tool-agnostic approach adaptable to local data capacity |
| **JA-CHRODIS [4,19,20,21]** | *Clinical guidelines; implementation evaluation* | Multimorbidity pilots not sustained after project funding ended in weaker governance contexts | **C3** | IPS-level institutional MM committee with assigned resources; designed for sustainability without dependence on external project funding |
| **SELFIE [23]** | *Scoping review* | Heterogeneous IT capacity; limited governance formalisation across European integrated care programmes; variable stratification approaches | **C1, C3, C5** | Tool-agnostic stratification in C1; governance formalisation at provider level in C3; graduated technology model in C5 |
| **Polypharmacy reviews [35]** | *Scoping review* | Medication management rarely embedded in MM-specific care plans in LMICs | **C2** | Medication reconciliation mandated as core clinical process step; deprescribing review integrated into C4 follow-up protocols |
| **OECD / Yordanov [36,45]** | *International policy document; observational study* | Financial incentives for integrated care underdeveloped in fragmented Latin American health systems | **C3** | Monitoring indicators in Table 4 designed to support future EPS performance-based contracting (medium-term objective) |
| **Endalamaw et al. [5]; Bhatt et al. [32]** | *Systematic review; scoping review* | Limited evidence on care model components in LMICs; barriers to person-centred multimorbid care in primary health systems | **C1, C2, C3, C4** | IPAM components address each identified implementation domain; design feasibility validated through expert consultation |
| **Oliveira et al. [33]** | *Systematic review* | Risk stratification tools show limited real-world implementation evidence in primary care; heterogeneous results across settings | **C1** | Tool-agnostic approach adopted; minimum-viable criteria allow implementation without requiring validated population-level stratification tools |
| **Cross-study synthesis — present study [5,7,27,32]** | *Systematic review [5]; programmatic report [7]; observational study [27]; scoping review [32]* | Reconciliation conceptually ambiguous; ICP absent from standard documentation; informal interprofessional communication; follow-up variability across Colombian IPS contexts | **C2, C3, C4** | C2 specifies reconciliation as a distinct documented clinical procedure; C3 formalises case conferences; C4 institutionalises proactive follow-up as standard workflow |
| **Cross-study synthesis — present study [7,13,27]** | *Programmatic reports [7,13]; observational study [27]* | Administrative processes disrupt care continuity despite strong clinician–patient relationships | **C3, C5** | C3 specifies direct patient–IPS communication channels; C5 Phase 1 enables appointment facilitation tools |

*ICP = individualised care plan; MM = multimorbidity; WB = World Bank; LMIC = low- and middle-income country; MDT = multidisciplinary team; EPS = health insurance entity (Entidad Promotora de Salud); IPS = institution providing health services (Institución Prestadora de Servicios de Salud); PHC = primary health care; ACG = Adjusted Clinical Groups; NGT = Nominal Group Technique.*
